# Intracardiac Placement of Needle Thoracostomy Catheter by Paramedics Due to Anatomic Variation: A Case Report

**DOI:** 10.5811/cpcem.63641

**Published:** 2026-10-06

**Authors:** Brian Strain, Scott Meeker, W. Seth Dukes, Paul Brian Savino

**Affiliations:** * Loma Linda University Medical Center, Department of Emergency Medicine, Loma Linda, California; † Arrowhead Regional Medical Center, Department of Emergency Medicine, Colton, California

**Keywords:** needle thoracostomy, tension pneumothorax, intracardiac, paramedic, case report

## Abstract

**Introduction:**

Needle thoracostomy is the only procedure in the United States for paramedic treatment of suspected tension pneumothorax within the most recent emergency medical services (EMS) national scope of practice model. We present a rare complication of an out-of-hospital left needle thoracostomy performed by paramedics after intracardiac placement of the catheter. The patient’s imaging studies in the receiving emergency department (ED) confirmed that the needle thoracostomy insertion location was correct, but the catheter entered the heart due to the patient’s existing atypical anatomy.

**Case Report:**

A 64-year-old unrestrained male driver in a roll-over motor vehicle accident was found in respiratory distress with difficulty auscultating left-sided lung sounds, left chest wall pain on palpation, and confusion. The first-arriving paramedics performed a left needle thoracostomy for suspected tension pneumothorax in advance of helicopter EMS transport. However, pulsatile blood flow from the catheter suggested intracardiac placement, which was confirmed on multiple imaging modalities in the ED. Imaging showed the catheter in the anterior second intercostal space at the midclavicular line. It was also revealed that the patient’s heart was prominently superior and nearly extended to the left chest wall with a majority of his left lung positioned posteriorly within the thorax. There were no signs of pneumothorax, hemothorax, subcutaneous emphysema, diaphragmatic injury, sternotomy wires, or other evidence that his atypical anatomy was the result of an exogenous process.

**Conclusion:**

Intracardiac placement of a needle thoracostomy catheter is a rare complication. In this case, the patient’s atypical anatomy contributed to asymmetric lung sounds and suspicion of tension pneumothorax. Regardless of which insertion site was chosen, the likelihood of intracardiac placement was potentially high due to the patient’s anatomy. Anatomical variation can cause lung sound asymmetry that mimics a pneumothorax, which emergency clinicians and paramedics must consider when assessing for tension pneumothorax.

## INTRODUCTION

Needle thoracostomy is the procedure authorized by the local emergency medical services (EMS) agency for prehospital treatment of suspected tension pneumothorax. This agency authorizes two sites of over-the-needle catheter insertion into the pleural space for decompression.^1^ The traditional site is anteriorly at the second intercostal space midclavicular line (ICS2-MCL). More recently, the axillary site at the fourth or fifth intercostal space (ICS4/5) at either the anterior axillary line or at the midaxillary line (MAL) has been adopted after studies indicated decreased chest wall thickness improved likelihood of reaching the pleural space.^2^ Prehospital pleural decompression in the United States is most often performed by needle thoracostomy.

There are alternatives to needle thoracostomy, such as a simple finger thoracostomy, in which a scalpel incision is made at the ICS4/5-MAL and the clinician bluntly dissects or spreads the tissue to access the pleural space with forceps or gloved finger sweeping the pleural space clear of air and/or blood. It is then left open to the atmosphere for continued decompression.^3^,^4^ Another alternative is a chest tube insertion. Both procedures are authorized by the local EMS systems and used only in limited settings by specially trained and qualified prehospital clinicians provided there is medical oversight and appropriate quality assurance.^5^

This case highlights a rare complication of an out-of-hospital needle thoracostomy due to the patient’s atypical anatomy mimicking a tension pneumothorax. The treating ground paramedics’ suspicion of intracardiac placement after insertion was confirmed at the receiving emergency department (ED). Imaging studies confirmed correct catheter insertion placement but revealed intracardiac extension due to the patient’s atypical anatomy.

## CASE REPORT

A 64-year-old unrestrained male driver with no known medical history presented to the ED via helicopter EMS (HEMS) for blunt traumatic injury after a roll-over motor vehicle accident. Per EMS, the patient was exhibiting respiratory distress with difficulty auscultating left-sided lung sounds, chest wall pain, and confusion. Initial vital signs were as follows: heart rate, 135 beats/min (bpm); oxygen saturation, 78% on room air; respiratory rate, 26 breaths/min; and initial Glasgow Coma Score (GCS) 10 (E2V2M6). Blood pressure was not attempted. The ground paramedic performed a needle thoracostomy using an 8-cm catheter in the left second intercostal space at the MCL for suspected tension pneumothorax. Immediately upon placement, pulsatile blood was noted from the catheter. The catheter was capped and left in place by the transporting HEMS team consisting of a flight paramedic and flight nurse.

Repeat vital signs en route included the following: heart rate, 128 bpm; blood pressure, 160/98 mm Hg; oxygen saturation, 94% on 15 L/min via nonrebreather mask; respiratory rate, 26 breaths/min; and GCS 14 (E4V4M6). The patient received fentanyl 200 mcg and ondansetron 4 mg intravenously en route as well as 1 g of tranexamic acid. The patient continued to have persistent diminished left-sided lung sounds on examination during transport.

Vitals obtained upon arrival to the ED revealed the following: blood pressure, 130/78 mm Hg; heart rate, 97 bpm; temperature, 97.9 °F; respiratory rate, 18 breaths/min; and oxygen saturation, 99% on 15 L/min via nonrebreather mask. The initial extended focused assessment with sonography in trauma exam, radiograph of the chest (Image 1), and computed tomography (CT) of the chest confirmed intracardiac placement of the thoracostomy catheter (Image 2). It was also noted that his heart was prominently superior and nearly extended to the left chest wall with most of his left lung positioned posteriorly within the thorax. Moreover, imaging did not show evidence of pneumothorax, hemothorax, or subcutaneous emphysema. The patient was taken to the operating room where it was again confirmed that the thoracostomy catheter penetrated the pericardium and the lateral left ventricle with subsequent small pericardial effusion without active hemorrhage. The catheter was removed, and the ventricle was repaired. The pericardium was left open, and a Jackson-Pratt drain was placed between the left atrium and left ventricle. Separately, a left-sided chest tube was placed intraoperatively.

*CPC-EM Capsule*

What do we already know about this clinical entity?

*Tension pneumothorax is an emergent cause of obstructive shock, treated by immediate pleural decompression.*

What makes this presentation of disease reportable?

*This presentation demonstrates how anatomic variation can mimic the presentation of tension pneumothorax in a trauma patient.*

What is the major learning point?

*Anatomic variation may distort lung sounds and mimic the clinical presentation of pneumothorax and tension physiology.*

How might this improve emergency medicine practice?

*Objective assessment, such as portable ultrasound, prior to needle decompression may clarify the diagnosis and prevent potential complications.*

The patient was admitted to the intensive care unit for ongoing evaluation and treatment of his injuries. Other significant injuries included unstable sixth cervical vertebra fracture with adjacent spinal epidural hematoma and first lumbar burst fracture. Planned surgical repair was deferred when the patient deteriorated because of progressive traumatic brain injury and sepsis. Based on the poor prognosis and the patient’s known wishes against life support, the family elected to proceed with palliative care.

## DISCUSSION

We present a case that highlights the potential iatrogenic injuries that can occur with needle thoracostomy in patients with anatomic variants. This is in contrast with previous reports showing complications secondary to the underlying traumatic injury causing distorted anatomy. In one such case, a patient was victim of a traumatic cardiac arrest following a motor vehicle accident with multisystem trauma. Helicopter EMS performed a left ICS2-MCL needle thoracostomy following return of spontaneous circulation and persistent hypotension without confirmation of a pneumothorax in the ED. The patient was later found to have an intracardiac needle thoracostomy.^6^ A retrospective study on prehospital needle thoracostomies identified a case of intracardiac placement. The study did not describe the details of placement; however, review of their CT image supplement revealed an intracardiac ICS2-MCL needle thoracostomy with likely mediastinal shift from visible large right-sided pneumothorax rather than inherent anatomic variation.^7^

Accurate detection and successful treatment of tension pneumothorax in the prehospital setting remains a challenge.^5^ Neeki et al emphasizes that traumatic tension pneumothorax is a clinical diagnosis with hallmarks of lung sound asymmetry and hypoperfusion in the setting of chest trauma.^4^ The study notes that the actual incidence of tension pneumothorax in the civilian population is not known. Contributing factors include an inability to differentiate iatrogenic pneumothorax caused by needle thoracostomy from pneumothorax resulting from pre-needle thoracostomy traumatic injury. The authors also indicate the difficulty of distinguishing clinical findings attributable to tension pneumothorax from other conditions in the prehospital setting.^4^ From review of this case we posit that atypical anatomy can also lead to false-positive identification of tension pneumothorax.

Various studies have demonstrated poor overall needle thoracostomy success rates relating to both insufficient catheter length and improper needle insertion location. Previously, the procedure was routinely performed using 14-gauge intravenous catheters up to 5 cm in length. Increasing the catheter length from 5 cm to 8 cm or greater was advocated after studies that showed many patients had a chest wall thickness greater than 5 cm.^2^,^5^,^8^ Over-the-needle 10 and 14 gauge/8cm l catheters are commercially available in pleural decompression kits. Changing from the anterior chest ICS2-MCL site to the axillary ICS4/5-MAL site was another strategy, given pectoral muscle mass thickness anteriorly.^2^,^4^,^5^ After one EMS agency changed its protocol to use the ICS 4/5-MAL as the primary site and increased catheter length to 9.5 cm in 2013, an analysis showed no significant difference in mortality outcomes when comparing cases in the preceding six years and three years after the change.^8^

Variability among clinicians’ ability to correctly locate the ICS2-MCL and the ICS4/5-MAL sites has been in reports of prospective simulation and in retrospective case reviews. Inferiorly positioned MCL catheter placement runs the risk of cardiac, diaphragmatic, or intraabdominal injury or placement. Diaphragmatic and intrabdominal placements are also a complication of inaccurate axillary site selection and insertion below the fifth ICS.^9^–^11^ In a study with 33 resident physician and medical student volunteers, the accuracy of correctly marking the ICS4/5 level along the axilla improved from 48% to 91% after brief instruction and addition of ultrasound guidance compared to visual inspection of physical landmarks and palpation alone.^9^ Novel approaches to address needle placement challenges now include commercially available template devices to place on the patient for ICS4/5-MAL site confirmation, as well as carbon dioxide detection devices that attach to the catheter to confirm pleural space entry.^12^,^13^ Prehospital use of portable ultrasound could have a role in both confirming tension pneumothorax and aiding in exclusion of tension physiology and reducing unnecessary needle thoracostomy insertions.^11^ Long-term study is needed to determine the extent of patient benefit and whether such strategies should be adopted as standard practice.

Many local EMS protocols require hypotension as evidence of tension physiology prior to performing needle thoracostomy. Many also include empiric bilateral decompression in cases of traumatic arrest with signs of chest trauma. The most recent Tactical Combat Casualty Care guidelines (January 2025) advocate early decompression without awaiting hypotension and advancing the needle/catheter fully with the hub at the skin using an 8-cm catheter.^11^ The most recent National Association of EMS Physicians’ statement on treatment of traumatic pneumothorax in the 2024 Prehospital Trauma Compendium warns of the increased risk of this technique in the civilian population with catheters longer than 7 cm given the difference in chest wall thickness.^5^ The discussion of anatomic variation affecting needle thoracostomy success has been limited to comparison of chest wall thickness. To our knowledge, there is no published research on how native variations in heart and lung position within the chest can impact needle thoracostomy outcomes.

Theoretically, tension physiology should shift vital structures away from the access site on the side of the pneumothorax. Without true tension physiology or when an alternative pathology explains the presentation, needle thoracostomy fails to provide therapeutic pleural volume expansion or protective anatomical shifts. Notably, inadvertent intracardiac placement can occur with other decompression devices as well. One case report details the management of a 44-year-old female pedestrian struck by a vehicle suffering multiple injuries and large left pneumothorax. Decompression attempted via Seldinger technique and with a 14-French pigtail catheter resulted in the catheter transecting a branch of the coronary artery before coiling in the ventricle.^14^

The potential of finger thoracostomy as an alternative to needle thoracostomy for paramedics in the United States is being studied. A retrospective propensity-matched cohort study of 34 patients in Texas suggested higher decompression success by ground paramedics with finger thoracostomy compared to needle thoracostomy. However, because the study was small and not generalizable, it does not support change to finger thoracostomy as the primary method for prehospital pleural decompression.^15^ There continues to be wide variability in needle thoracostomy success as well as a high incidence of overtriage of possible tension pneumothorax resulting in needle thoracostomy attempts that are not clinically indicated.^4^,^5^ Thus, a generalizable head-to-head prospective randomized control trial study of needle thoracostomy to finger thoracostomy may not be feasible, setting aside current prehospital scope of practice model limitations.

## CONCLUSION

The occurrence of tension pneumothorax in the civilian population remains statistically low. Thus, a paramedic may not encounter a true case of tension pneumothorax during their entire career.^5^ Questions remain regarding how to reduce procedural error in the prehospital setting for high-risk, low-frequency and yet potentially life-saving procedures like needle thoracostomy. The lessons of this case may support efforts to include additional assessment technologies such as portable ultrasound and other novel approaches to improve accurate tension pneumothorax recognition and treatment and reduce iatrogenic harm in the prehospital setting.

## Figures and Tables

**Image 1:**
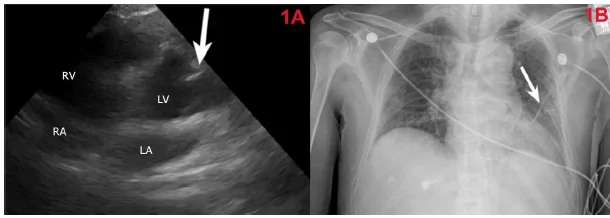
(1A) Initial subxiphoid ultrasound image showing catheter tip in the left ventricle of the heart (arrow). (1B) Initial chest radiograph showing the catheter in midclavicular position overlying the cardiac silhouette (arrow). *LA*, left atrium; *LV*, left ventricle; *RA*, right atrium; *RV*, right ventricle.

**Image 2:**
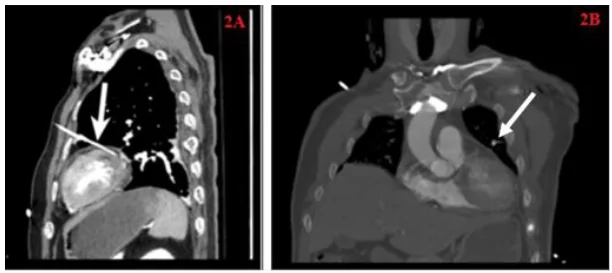
(2A) Sagittal image of chest computed tomography (CT) at the approximate left midclavicular line with the catheter (arrow) traversing from entry at the second intercostal space to the superior aspect of left ventricle. (2B) Coronal image of chest CT showing needle thoracostomy insertion at the second intercostal space along the midclavicular line (arrow). The heart is adjacent to the chest wall where needle insertion at the fourth or fifth intercostal space at the midaxillary line would also likely result in iatrogenic injury and/or intracardiac placement.
